# An Observational Longitudinal Study Investigating the Effectiveness of 75 mg Pregabalin Post-Hemodialysis among Uremic Pruritus Patients

**DOI:** 10.1038/srep36555

**Published:** 2016-11-08

**Authors:** Tahir Mehmood Khan, David Bin-Chia Wu, Bey-Hing Goh, Learn-Han Lee, Abdul Aziz Alhafez, Syed Azhar Syed Sulaiman

**Affiliations:** 1 School of Pharmacy, Monash University, Bandar Sunway, Jalan Lagoon Selatan, 46700 Selangor, Malaysia; 2 College of Clinical Pharmacy, King Faisal University, Alahsa, Saudi Arabia; 3 Director and Senior consultant, Aljaber Kidney and Dialysis Center, Alahsa, Eastern Province, Saudi Arabia; 4 School of Pharmaceutical Science, University Sains Malaysia Pulau Pinang, Malaysia

## Abstract

A prospective, observational, longitudinal study was conducted to assess the effectiveness of 75 mg pregabalin (PG) post-hemodialysis (pHD) for treatment-resistant uremic pruritus (UP). A total of forty-five patients completed the entire six week follow-up. At the baseline assessment, the majority of the patients were distressed by the UP frequency and intensity. Sleep (mean = 3.30 ± 1.1), leisure/social activities (mean = 2.90 ± 0.80) and distribution (mean = 2.92 ± 0.34) were the three domains that were primarily effected by the UP. Overall, further reduction in the 5D-itching scale (IS) was noted at day 42, which confirmed a sustained (B = −12.729, CI −13.257 to −12.201, p < 0.001) relief of pruritus severity among patients with treatment-resistant pruritus. Patients with a higher serum calcium level had a score difference of +1 from the other patients (B = 1.010, p = 0.061). There was a reduction of 12 points compared to the baseline 5D-IS for each patient on day 42 after using pregabalin 75 mg PD pHD for 42 days, which represented major relief. Among the demographic factors, only gender was significantly associated with the 5D-IS score.

Earlier data from 1973 showed a very high incidence of uremic pruritus (UP) among end-stage renal disease (ESRD) patients. In some studies, the incidence was reported to be 90% of this population^1^. However, the incidence seemed to reduce to 60–70% by the mid-1980s, perhaps due to a better understanding of the disease pathogenesis^2^,^3^,^4^. Recent studies reported a variable incidence of UP among the study population that ranged from 40–70%^5^,^6^,^7^,^8^. Thus, damage to the skin among ESRD patients receiving dialysis or renal replacement therapy is most likely due to UP. In addition to these anatomical consequences, UP has a major impact on the sleep quality and the psychosocial and social wellbeing of the patients^9^,^10^,^11^. With continuous research in the basic health sciences, the UP prevalence has declined compared to the past. However, UP remains a major clinical symptom and is often a medical challenge in severe cases due to the complex processes involved in the generation of UP stimuli.

Recently, the use of neuroleptic agents, such as gabapentin and pregabalin (PG), has massively increased for the management of treatment-resistant UP. Neuroleptic agents such as gabapentin have good effectiveness for the treatment of pruritus that is resistant or non-responsive to topical and other systemic treatment options^12^. However, pregabalin (PG) has been found to be effective in providing relief for the patient’s condition in some situations where patients are unable to tolerate gabapentin^13^,^14^. Gabapentin and PG have drastic differences in their pharmacokinetics and pharmacodynamics. The oral absorption of gabapentin is slower than PG, which attains its plasma peak concentration within 1 hour^15^. Furthermore, the dose-dependent concentration increase is not a characteristic of gabapentin. However, the bioavailability of gabapentin reduces from sixty to 33.0% when the dose increases from 900 mg/day to 3600 mg/day^16^. For PG, the bioavailability remains more that 90.0% regardless of the dose increase. These pharmacokinetic benefits provide a pharmacodynamics edge to PG over gabapentin.

However, gabapentin is often not tolerated by ESRD patients due to their compromised renal functions, poor oral absorption and the low bioavailability of gabapentin, which is another challenge. Therefore, the use of higher gabapentin doses for ESRD patients has led to an increased risk of adverse events associated with its use^14^. PG is superior to gabapentin in this regard. A recent longitudinal study conducted by Shavit *et al*. (2013) reported the therapeutic effectiveness of PG at a dose of 25–50 mg/day among uremic patients with treatment-resistant pruritus who had shown resistance to antihistamines and emollients^17^. Additionally, Aperis *et al*. (2010) and Rayner *et al*. (2013) reported the effectiveness of PG at a dose of 25 mg/day among ESRD patients with treatment-resistance pruritus^13^,^18^, with the severity of the pruritus markedly reduced among the patients treated with PG. However, a critical analysis of the results reflected the need for strong methodological studies. Most studies advocated the daily use of 25–50 mg/day to manage UP. However, administering a single dose of 75 mg/day post-HD (three times a week, 75 mg PG) has not been investigated. PG is cleared through renal excretion; thus, a single PG dose of 75 mg/day post-HD (pHD) may be effective. If patients receive a 25 or 50 mg dose of PG once or twice daily, the likelihood of an adverse event will be double due to the repetitive exposure. Conversely, a single 75 mg PG dose after each dialysis session (3 times a week, 75 mg PG) may have a better therapeutic outcome due to the higher peak effect and single dose administration, which is more convenient for the patients. The aim of the current study was to assess the therapeutic effectiveness of 75 mg PG pHD for treatment-resistant UP among ESRD patients.

## Materials and Methods

A prospective observational study design was planned to estimate the efficacy, tolerability and safety of PG among ESRD patients with treatment-resistant UP. This study was conducted at Al-Jaber Kidney Center (AJKC) in the eastern province of Al-Ahsa (AH) in Saudi Arabia. At the selected study center, 75 mg PG post-hemodialysis is one of the recommended regimens to manage the severity and frequency of pruritus among ESRD patients.

### Study duration and data collection

The duration for this study was from April 1, 2012, through May 29, 2013. A structured data collection form was used to record patient demographics and all subjective information. Furthermore, all laboratory parameters and vital signs were recorded. The Arabic version of the 5D-IS was used to quantify the UP^19^. The 5D-IS score was calculated at four different intervals to measure the effect over time. The assessment plan is shown in Table 1.

### Study population

A total of 314 patients are registered for dialysis at the AJKDC. These patients are managed in three shifts (morning, afternoon and evening). Of the total number, 173 are male patients and 112 are female patients. A total of N = 285 patient are on HD, whereas the rest are on peritoneal dialysis. All the hemodialysis patients were assessed for the severity and frequency of pruritus.

### Standard treatment protocol for UP at AJKC

At AJKC, any ESRD patient suffering from UP is treated using loratadine (10 mg daily) for at least two months alone or in combination with Vaseline lotion as an emollient (first line treatment). However, if the severity of UP is not reduced, 75 mg pregabalin is administered pHD to manage the refractory or treatment-resistant UP (second line treatment).

### Study population and study size

Patients non-responsive to the first line treatment were considered for potential inclusion in the study. The minimum effective sample size for this study was estimated using a significance level of 5% and a power of 90%^20^ using the following equation.


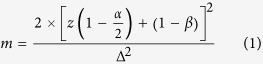


where z(1 − α/2) and z(1 − β) represent percentage points of the normal distribution for the statistical significance level and power, respectively, and Δ represents the standardized difference (the treatment difference divided by its standard deviation). We assumed that if a patient’s 5D-IS score was 20 prior to administration of 75 mg PG, at the end of study the score should be reduced by eight points with an assumed standard deviation of 10. Thus, for a patient who was ranked with severe itching, a mean reduction of eight points in the score would result in his or her itching becoming minor or moderate [Score = 10–15 (mild itching), 16–20 (moderate itching) and 21–25 (severe itching)]. The assumed reduction in the mean score and the standard deviation were used to calculate the effect size using the following equation:


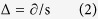


where ∂ is the mean reduction in the 5D-IS score and s = the standard deviation.

In the case of the current study, ∆ = 0.8


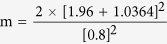


Using the values from the table for a significance level of 5%, z (1 − α/2) = 1.96, and a power of 85%, z (1 − β) = 1.0364. Based on equation 1, the minimum required sample size for this study was 33.

### Ethical considerations and data analysis

Informed written consent was collected from all participants. A native Arabic speaker explained the purpose of their participation in the study and the consent statement. The study protocol/experimental protocols were approved by the “Research Ethics Committee” operated under the continuous medical education and research department at King Fahad Hospital and the director of the AJKC. Moreover, experimental protocols were also approved by the deanship of scientific research, King Faisal University, eastern province, Saudi Arabia. Moreover, all methods were carried out in accordance with relevant guidelines and regulations of the AJKC. AJKC is one of the affiliated dialysis centers of King Fahad Hospital, and operated under Ministry of Health, Saudi Arabia. The data were coded into nominal, ordinal and continuous variables using SPSS^®^ version 20 where applicable. Both the descriptive and inferential statistics were applied to attain the purpose of the study As the effect of PG in itching was longitudinal, i.e. multiple measurements were taken within each subject at different time points and the data was clustered in to subgroups, the traditional regression model e.g. general linear model is inappropriate as it assumes independence among observations at different time points. If correlation structure is not considered, the likelihood to have a wrong estimation of regression model parameters will be^21^,^22^. Thus, Harrison and Hulin (1989) recognized generalized estimating equations (GEEs) as an appropriate assessment tool for the analysis of longitudinal data^23^. GEEs was developed by Liang and Zeger (1986) and proved to provide more reliable regression estimation for the longitudinal data or repeated measures^24^,^25^,^26^,^27^,^28^. Therefore, to estimate the efficacy of PG in each individual case, Generalized Linear Model based (working correlation matrix AR (1) on GEEs was used to estimate the improvement over the time in each patient who was assessed at four occasions. Covariates (i.e., smoking habit, gender, history of dialysis and lab values) were included to control for potential confounding effects. Wald chi-squared test was used to test the significance of regression coefficients. Statistical significance level was considered significant at 0.05 with the confidence interval of 95.0%.

## Results

Upon the initial assessment, fifty-three patients were found to be the potential candidates for PG therapy. Two patients who missed the baseline assessments and performed five dialysis sessions at another dialysis center were excluded. Therefore, the final number of patients included across the study duration was n = 51. Overall, the majority of the patients (70.6%) were male and had a mean age of 55 years. Additionally, approximately 19 (37.3%) of the patients were active smokers. Dialysis for all patients was performed at ≤1.5 Kt/V. The demographic information of the selected patients after safety screening is shown in Table 2.

Most of the patients received Alfacalcidol (1.25 mcg qd) during the study duration and heparin (2000 units/pHD). Details about the other common medications administered to the patients during the study period are provided in Table 3.

### Baseline assessment at day 0

The baseline assessment for the fifty-one patients included in the study was performed at day 0 using the 5D-IS. The majority of the patients were distressed by the frequency and intensity of UP. Overall, the duration, degree and direction were the three main domains affected by UP. However, disabilities in sleep (mean = 3.30 ± 1.1) and leisure/social activities (mean = 2.90 ± 0.80) were the two areas most effected by UP. Overall, the mean score at the baseline assessment was 19.1 ± 1.7 [range 16–23]. Details are provided in Table 4.

On day ten, two male patients from the 71–80 year age group complained about blurred vision. On day thirteen, three male patients from the same age group reported somnolence. One female patient from the 41–50 year age group complained of dizziness on day thirteen and was discontinued from PG. These six patients were dropped from the overall analysis and the relevant data was also excluded from the final data analysis.

### Comparison of the effect of PG (75 mg pHD) on days 0, 14, 28, and 42

After excluding the six patients, a total of forty-five patients completed the entire six week follow-up. Upon assessment at day twenty-eight (5D-IS = 7.7 ± 1.2), there was a massive reduction in the severity and intensity of UP. Further improvement was also noted at day forty-two (5D-IS = 6.49 ± 0.90). Details are shown in Table 4.

### Longitudinal assessment of the effect of PG and the change in the 5D-IS over time

Upon transforming the 5D-IS result (days 0, 14, 28 and 42) in to longitiudal data, GEEs were applied using time as an independent variable and 5D-IS as a dependent variable. To control for potential confounders, other variables, including gender, age, serum phosphorus level, serum parathyroid hormone, serum creatinine, serum calcium and uric acid, were added in the model to test their associations with the increase or decrease in the 5D-IS score. The effects of the dependent variable (score) and other variables in the model were estimated using the “Wald Chi square” (18.445, df = 1 and p < 0.001). We observed that the overall improvement in the 5D-IS over time was significant, which confirmed the effective of PG in reducing the intensity and severity of itching over time. The baseline assessment was kept as zero to compute the reduction over time. At day 14, there was a reduction of 0.280 in the patients’ 5D-IS scores (p = 0.032) (Table 5). However, at day 28, the reduction in the 5D-IS score was 11.475 for each patient. Moreover, the reduction in the score was more than the reduction we assumed when calculating the sample size. At day 42, a further reduction in the 5D-IS was noted, thereby confirming a sustained relief for the severity of pruritus among patients with treatment-resistant pruritus. The details are provided in Table 5.

Moreover, a comparison of the lab results between the baseline assessment and day 28 revealed that uric acid, serum calcium, serum phosphate, ALT, and the iron binding capacity had significant differences between the two time points (Table 6). Based on the analysis shown in Table 5, it was difficult to conclude the association of lab values with the severity and intensity of UP. Upon further analysis, patients with higher serum calcium levels were found to have a score difference of +1 compared to the other patients (B = 1.010, p = 0.061). Moreover, a reduction in serum urea and serum phosphate was identified as the two factors that reduced the severity and intensity of UP, although the association was not significant (Table 7).

We also demonstrated that smokers were likely to have higher scores (+0.097) than non-smokers (B = 0.097, p = 0.739), but this difference was not significant. Overall, every male patient was likely to have a slightly higher 5D-IS score (+0.102) than the female patients (B = 0.102, p = 0.032), and this difference was significant. Patients with a disease history of 5–6 years were more likely to have a higher 5D-IS score (+0.612) (B = 0.612 CI −0.221–1.444) than the other patients. However, patients with a disease history of more than six years had a comparatively lower 5D-IS than those with a disease history of six and less than six years. The details are shown in Table 7.

### Side effects associated with the use of pregabalin

Three patients complained of slight dizziness (especially the evening of the day they were administered PG) at day twenty-eight but were willing to continue the treatment. At day twenty-eight, two patients reported blurred vision, and three more patients reported the same symptom on day forty-two. Somnolence was reported by five patients (day 42) from the 51–60 year age group, especially during the evening on the day they were administered PG. One patient reported dry mouth on day twenty-eight, and six patients reported constipation.

## Discussion

The current study may be the first methodologically established longitudinal study to estimate the effect of pregabalin over time. In contrast, previous studies that assessed the use of pregabalin for the treatment of UP and neuropathic pruritus among ESRD patients used 25–50 mg pregabalin per day to treat UP^13^,^17^,^29^. The PG dosing used in this study (75 mg pHD or 75 mg PG three times a week) gave superior results to those of an earlier crossover study that reported relief among ESRD patients suffering from neuropathic pruritus with a dose of 75 mg/day^14^. Additionally, a recent randomized clinical trial strengthened the results of this study by administering 75 mg PG twice a week among patients suffering from UP^30^. Based on these references, PG is an effective regimen for ESRD patients suffering from UP and neuropathic pruritus. However, the current study was the first to test the effect of 75 mg pregabalin PHD among UP patients. Based on the dialysis frequency (three times per week), each patient was administered an estimated dose of 33 mg per day. Compared to the baseline assessment, at day 14 the reduction in the score for every patient was B = −0.280 (p = 0.032). It is difficult to explain why the response in the 5D-IS score was not more prominent on day 14; however, a mild improvement was noted in the disability domains (house work/errands and work/school). Perhaps due to the nature of the study design and assessment schedule, it was not possible for the current study to identify the reason for this minor decline in the score at day 14. However, the results of current study are consistent with the day 14 results from Yue, J *et al*.^30^, which reported a slight to negligible decline in the VAS among patients receiving PG. Future studies should consider assessing the severity of UP after each dialysis session, which may be inconvenient for the patients but will assist in pinpointing the time window during which pregabalin gives the maximum response. Nevertheless, the improvement was prominent at day 28 (p < 0.001), and the reduction in each patient’s score was B = −11.475 (CI −12.019 to −10.932) compared with the baseline 5D-IS score. Further significant improvement was also noted at day 40 (B = −12.729 (CI −13.257 to −12.201, p < 0.001). Additionally, most of the patients expressed that they were happy with the treatment outcome on days 28 and 42, even though it was not the objective of the current study to determine patient satisfaction with the treatment. In some cases, the patients decided to continue the treatment even in the presence of mild to moderate side effect. Perhaps dose tapering or a reduction will be beneficial for reducing the severity of adverse events, especially when a notable decline in the severity and frequency of UP is observed. Overall, by addressing the effect of PG, the current study confirms the results of previous studies that have reported the effectiveness of pregabalin among ESRD patients with UP and neuropathic pruritus^13^,^14^,^17^,^29^,^30^,^31^. Moreover, the current study has provided new evidence for the use of 75 mg PG (three times a week) to manage UP among ESRD patients who are non-responsive to other treatments.

### Association of demographic factors with UP

To date, there is a scarcity of epidemiological data exploring the incidence of UP based on ESRD patient demographic data. Some self-reported surveys from Europe reported a higher incidence of itching among healthy women^32^. Among most of the studies conducted on UP patients, the number of male patients was higher than the number of female patients^7^,^13^,^14^,^17^,^29^,^31^,^33^. However, keeping in mind the results of current study, it was difficult to conclude whether the ESRD incidence was higher among the male patients or whether UP was more prevalent among men. This factor was recently explored by a Japanese epidemiological study that investigated the incidence, etiology and prognosis of UP and its association with different variables^34^. In the current study, treatment resistance to UP was more frequent among men than among women. At the baseline assessment, gender was found to be a significant factor (and the only item) associated with the 5D-IS score (*x*^2^ = 13.922, df = 7, p = 0.033, CI 0.028 to 0.38).

Addressing the situation throughout the duration of study, the male patients had a significantly higher score than the female patients (B = 0.102, CI −0.498 to 0.720, p = 0.032). Additionally, smokers had a higher 5D-IS score than non-smokers (Table 5). Based on the existing literature on UP, it is impossible to justify how and why smokers have a higher intensity of itching. Future studies should explore this issue in detail; it is possible that a central effect of nicotine may have some association with the pruritus severity.

### Association of the ESRD history and laboratory values with the UP intensity and severity

The UP severity is associated with high serum phosphorous, calcium, blood urea nitrogen and parathyroid hormone (PTH) levels^35^,^36^,^37^. A high PTH level has a major effect on the serum phosphorous and calcium levels. Moreover, PTH induces inflammatory pathways by activating mast cells, which is why PTH is often reported as a potential factor that promotes and aggravates UP among ESRD patients^37^. Overall, there is a understanding that a persistent high level of blood urea nitrogen has a major impact on the severity and intensity of UP^37^. A minor reduction in the blood urea nitrogen level resulted in a reduction in UP (B = −0.053, p = 0.041). Moreover, patients with a high uric acid level had a slightly higher 5D-IS score (B = 0.007, p = 0.002). A higher score was also observed for patients with a higher serum calcium level, although the association was not significant. Various studies have shown a significant association between a high level of calcium and the UP severity^35^,^36^,^37^. Apart from these laboratory values, a history of ESRD was found not significantly associated with the UP severity. However, patients on dialysis for 5–6 years had a higher 5D-IS score than the other patients.

## Limitations

One limitation in this study was that we assessed UP only at specific time points and not on a daily basis. Nevertheless, the patients were interviewed by the researcher upon receiving dialysis to assess the presence or absence of UP. Additionally, no plasma concentrations were examined to confirm the results. This study is not a randomized clinical trial; therefore, the authors could not determine whether the patients used topical emollients that might have added effects on their conditions. Although the use of a smaller sample size from the Arab population may limit the generalizability of results, the use of a repeated-measures design eliminates the individual differences that occur between subjects and reduces biological variability, which increases the power of the results. Additionally, an appropriate sample size calculation was performed to ensure that the current study with 45 patients was sufficient to achieve the desired statistical power of 85%, which was unfortunately missing in previous studies. Finally, the lack of a comparator is another issue that may hinder the ability of researchers to estimate the comparative effect versus the placebo or any other therapeutic regimen. Future studies may consider assessing the effect of pregabalin (75 mg pHD) in a multicenter setting with a larger population size to obtain more conclusive results.

## Conclusion

Upon administration of 75 mg pregabalin after hemodialysis, a significant reduction in the UP score was seen at day 28 compared to the 5D-IS score at the baseline assessment on day 0. There was a reduction of 12 points in the 5D-IS for each patient on day 42 compared to the baseline, which represented major relief after the use of pregabalin 75 mg (pHD) for 42 days. Gender was the only demographic factor that was significantly associated with the 5D-IS score.

## Additional Information

**How to cite this article**: Khan, T. M. *et al*. An Observational Longitudinal Study Investigating the Effectiveness of 75 mg Pregabalin Post-Hemodialysis among Uremic Pruritus Patients. *Sci. Rep.* **6**, 36555; doi: 10.1038/srep36555 (2016).

**Publisher’s note:** Springer Nature remains neutral with regard to jurisdictional claims in published maps and institutional affiliations.

## Figures and Tables

**Table 1:** Assessment plan for study outcome.

Day	Assessment of pruritus with 5D-IS	Other assessment
0	Baseline assessment	Patient risk assessment and baseline 5D-IS
14	Initial assessment	Comparative assessment to baseline 5D-IS
28	Assessment for pruritus relief	Improvement in pruritus
42	Final Assessment	Sustainability of PG effect

**Table 2:** Patient demographic information n = 51.

Demographic Variables	N (%)
Age	
*Median* = *55 years*	
30 to 40 years	7 (13.7%)
41 to 50 years	13 (25.5%)
51 to 60 years	20 (39.2%)
61 to 70 years	6 (11.8%)
71 to 80 years	5 (9.8%)
Gender	
Male	36 (70.6%)
Female	15 (29.4%)
History of Renal Disease	
*Median* = *4.0*	
1 to 4 years	34 (66.7%)
5 to 6 years	12 (23.5%)
>6 years	5 (9.8%)
Smoking Habit	
Active smokers	19 (37.3%)
Non-smokers	32 (62.7%)
Education	
Primary	17 (33.3%)
Secondary	7 (13.7%)
College/High school	15 (29.4%)
Islamic education	12 (23.5%)
Job Status	
Jobless	17 (33.3%)
House wife/stay at home	7 (13.7%)
Own business	1 (2.0%)
Private company Job	3 (5.9%)
Government Job	20 (39.2%)
Farm/Agriculture job/business	3 (5.9%)
Marital Status	
Married	48 (94.1%)
Single	1 (2.0%)
Divorced	1 (2.0%)
Widowed	1 (2.0%)
Dialysis Adequacy	
≤1.5 Kt/V	51 (100.0%)
Dialysis Frequency	
3 times a week/4 hrs per session	51 (100.0%)

*Note: liquid acid concentrate (1* + *44 AC-F*) *was used as a dialyzer after mixing with bicarbonate concentrate (8.4*%) *and purified water.*

**Table 3:** Medications consumed by the patients.

Drug	Dose Frequency	N (%)
Alfacalcidol	1.25 mcg qd	51 (100.0%)
Heparin	2000 units/pHD	51 (100.0%)
Omeprazole	20 mg/qd	37 (72.2%)
	40 mg mg/qd	3 (5.9%)
Paracetamol	1 gm qd	17 (33.3%)
Lactulose	10 ml PO bid	16 (31.4%)
	15 ml PO qd	1 (2.0%)
Allopurinol	100 mg/qd	7 (13.8%)
Pentoxifylline	400 mg PO bid	6 (11.8%)
Ventolin	3 puff q 4–6 hrs	5 (9.8%)
Caco3	600 mg bid	25 (49.0%)
	600 mg tid	26 (51.0%)
Sevelamer	800 mg tid	40 (78.4%)
	1.6 gm tid	11 (21.6%)
Cinnacalcate	30 mg bid	2 (3.9%)
	30 gm qid	18 (35.2%)
	60 mg bid	6 (11.8%)
	90 mg bid	2 (3.9%)
Calcium Resonium	15 gm tid	10 (19.6%)

**Table 4:** 5D-IS assessment results on days 0, 14, 28 and 42 (n = 45).

5D-IS domains	Mean score at baseline assessment Day 0	Mean score at baseline assessment Day 14	Mean score at baseline assessment Day 28	Mean score at baseline assessment Day 42
Duration	3.86 ± 0.74	3.96 ± 0.69	1.4 ± 0.49	1.13 ± 0.34
Degree	4.41 ± 0.60	4.0 ± 0.73	1.3 ± 0.46	1.07 ± 0.27
Direction	4.27 ± 0.75	4.31 ± 0.73	1.4 ± 0.50	1.35 ± 0.48
Disability				
Sleep	3.30 ± 1.1	3.30 ± 1.06	1.3 ± 0.46	1.0 ± 0.0
Leisure/Social Activity	2.90 ± 0.80	2.90 ± 1.02	1.8 ± 0.65	1.6 ± 0.48
House work/errands	1.90 ± 0.66	1.80 ± 0.63	1.3 ± 0.48	1.3 ± 0.47
Work school	1.80 ± 0.83	1.74 ± 0.80	1.43 ± 0.47	1.39 ± 0.49
Distribution	2.92 ± 0.34	2.92 ± 0.34	1.7 ± 0.54	1.07 ± 0.71
Mean 5D-IS score	19.1 ± 1.7	18.8 ± 1.7	7.7 ± 1.2	6.49 ± 0.90
Range	16 to 23	16 to 22	6 to 11	5 to 8

**Table 5:** Reduction in 5D-IS over time in each patient (n = 45).

Time	B	CI [95%]	Wald Chi-Square	df	p value
Day 0	0^a^	—	—	—	—
Day 14	−0.280	−0.535–−0.024	4.612	1	0.032*
Day 28	−11.475	−12.019–−10.932	1712.067	1	<0.001*
Day 42	−12.729	−13.257–−12.201	2234.232	1	<0.001*

^a^Set to zero because this parameter is redundant.

^*^Significant. p < 0.05 was considered significant.

**Table 6:** Comparative analysis of lab values on days 0 and 28 (n = 45).

Lab test	Baseline assessment Mean ± SD	Assessment at Day 28Mean ± SD	Z	p
Blood urea nitrogen				
(BUN)	24.5 mmol/L ± 7.6	23.9 mmol/L ± 5.9	−0.056	0.955
Uric acid	685.9 mg/dL ± 71.9	610 mg/dL ± 65.1	−3.070	0.002*
Serum Creatinine	1021.7 μmol/L ± 310	950.5 μmol/L ± 245	−1.733	0.083
Calcium	2.37 mmol/L ± 0.30	2.46 mmol/L ± 0.20	−3.051	0.002*
Chloride	100 mmol/L ± 2.0	101 mmol/L ± 1.4	−0.939	0.347
Potassium	4.9 mmol/L ± 0.8	4.7 mmol/L ± 0.61	−0.774	0.439
Sodium	138.1 mmol/L ± 3.6	139.4 mmol/L ± 3.6	−2.552	0.011*
Para Thyroid				
Hormone	66.5 pg/ml ± 42.0	55.05 pg/ml ± 39.5	−1.233	0.218
Serum Phosphate	1.97 mmol/L ± 0.46	2.16 mmol/L ± 0.40	−3.647	<0.001*
Fasting blood glucose	9.97 mg/dL ± 3.5	9.91 mg/dL ± 3.62	−0.532	0.595
SGOT (AST)	27.9 IU/L ± 7.3	27.6 IU/L ± 7.8	−0.236	0.813
SGPT (ALT)	24.8 IU/L ± 9.2	25.2 IU/L ± 7.7	−2.139	0.032*
Cholesterol	268.6 mg/dl ± 24.7	259.6 mg/ dl ± 25.2	1.483	0.216
Serum Iron	11.7 mmol/L ± 5.18	12.0 mmol/L ± 3.6	−0.768	0.443
Iron Binding				
Capacity	436.0 μ/dL ± 185.0	444.0 μ/dL ± 121.1.0	−2.641	0.008*
Ferritin	378.7 ng/mL ± 68.3	384.2 ng/mL ± 276.1	−0.716	0.474
Albumin	33.3 gm/dl ± 6.8	32.8 gm/dl ± 3.08	−1.163	0.245
White blood cells	6.8 × 10^3^/cu mm ± 1.9	6.63 × 10^3^/cu mm ± 1.5	−0.589	0.556
Red Blood corpuscles	4.13 × 10^6^/cu mm ± 0.35	4.16 × 10^6^/cu mm ± 0.34	−0.197	0.884
Hemoglobin	11.2 g/dl ± 0.35	11.3 g/dl ± 2.21	−1.414	0.157
Hematocrit (%)	34.8% ± 4.9	35.5% ± 5.2	−0.261	0.794
Mean corpuscular volume	80.2 ± 7.6	80.8 ± 5.6	−0.145	0.885
Platelets	232.3 × 10^3^ per mm^3^ ± 44	222.3 × 10^3^ per mm^3^ ± 37.5	−1.960	0.051

Wilcoxon signed rank test was applied, p-value less than 0.05 was considered significant.

**Table 7:** Effect of other covariates on the 5D-IS score over time (n = 45).

	B	CI [95%]	Wald Chi-Square	df	Sig.
Smoking					
Smoker	0.097	−0.477–0.672	0.111	1	0.739
Non-smoker	0^a^				
Gender					
Male	0.102	−0.498–0.720	0.111	1	0.032*
Female	0^a^				
History of Renal disease					
1 to 4 years	0.167	0.444–0.777	0.287	1	0.592
5 to 6 years	0.612	−0.221–1.444	2.072		0.150
>6 years	0^a^	—	—		—
Blood urea nitrogen	−0.053	−0.103–0.002	4.193	1	0.041
Uric acid	0.007	0.003–0.011	9.828	1	0.002*
Serum creatinine	0.000	−0.001–0.001	0.292	1	0.589
Serum phosphate	−0.210	−0.778–0.358	0.526	1	0.468
Para thyroid hormone	0.002	−0.001–0.005	1.528	1	0.216
Serum calcium	1.010	−0.047–2.066	3.510	1	0.061
Number of dialysis session	0.001	−0.006–0.002	3.907	1	0.048

Generalized linear model was used based on GEE, using working correlation matrix AR(1).

^a^Set to zero because this parameter is redundant.

^*^Significant; p < 0.05 was considered significant.
